# Counter-Current Fractionation-Assisted Bioassay-Guided Separation of Active Compound from Blueberry and the Interaction between the Active Compound and α-Glucosidase

**DOI:** 10.3390/foods10030509

**Published:** 2021-03-01

**Authors:** Hongkun Xue, Xiaohan Zhu, Jiaqi Tan, Linlin Fan, Qian Li, Jintian Tang, Xu Cai

**Affiliations:** 1 Key Laboratory of Particle & Radiation Imaging, Ministry of Education, Department of Engineering Physics, Tsinghua University, No. 30 Shuangqing Road, Haidian District, Beijing 100084, China; xuehk@mail.tsinghua.edu.cn (H.X.); zhuxh18@mails.tsinghua.edu.cn (X.Z.); liq@mail.tsinghua.edu.cn (Q.L.); tangjt@mail.tsinghua.edu.cn (J.T.); 2 Academy for Advanced Interdisciplinary Studies, Peking University, No. 5 Yiheyuan Road, Haidian District, Beijing 100871, China; tanjiaq@pku.edu.cn; 3 Graduate College, Tianjin University of Traditional Chinese Medicine, 10 Poyanghu Road, West Area, Tuanbo New Town, Jinghai District, Tianjin 301617, China; Fanll@tutcm.edu.cn

**Keywords:** blueberry, bioassay-guided separation, cyanidin-3-glucoside, α-glucosidase, interactions

## Abstract

An efficient strategy for the selection of active compounds from blueberry based on counter-current fractionation and bioassay-guided separation was established in this study. Blueberry extract showed potential α-glucosidase inhibitory activity. After extraction by different solvents, the active components were enriched in water. The water extract was divided into six fractions via high-speed counter-current chromatography to further track the active components. Results indicated that the α-glucosidase inhibition rate of F4 was remarkable higher than the others. Cyanidin-3-glucoside (C3G) with a purity of 94.16% was successfully separated from F4 through column chromatography, and its structure was identified by ultraviolet spectral, Fourier-transformed infrared spectroscopy, high-performance liquid chromatography-electrospray ionization-tandem mass spectrometry, ^1^H nuclear magnetic resonance (NMR), and ^13^C NMR. The interaction mechanism between C3G and α-glucosidase was clearly characterized and described by spectroscopic methods, including fluorescence and circular dichroism (CD) in combination with molecular docking techniques. C3G could spontaneously bind with α-glucosidase to form complexes by hydrogen bonds. The secondary structure of α-glucosidase changed in varying degrees after complexation with C3G. The α-helical and β-turn contents of α-glucosidase decreased, whereas the β-sheet content and the irregular coil structures increased. Molecular docking speculated that C3G could form hydrogen bonds with α-glucosidase by binding to the active sit (Leu 313, Ser 157, Tyr 158, Phe 314, Arg 315, and two Asp 307). These findings may be useful for the development of functional foods to tackle type 2 diabetes.

## 1. Introduction

Natural products have very complex chemical composition. Traditional methods for the separation of active compounds are complicated, time-consuming, and inefficient. Thus, a potential method of bioassay-guided separation (BGS) was proposed to improve the efficiency of separating active compounds from natural plant resources [1,2]. By combining chromatography with biological activity detection, the active compounds were enriched to obtain high activity pure compounds [3,4]. Bioactive compounds, including antimicrobial coumarins, anti-lung cancer compound, and water-soluble antioxidants, have been successfully isolated by BGS [5,6,7]. Counter-current chromatography, as the most vital chromatographic technique in BGS, is often used for the separation of various active compounds from natural plant resources [8,9]. However, some trace active compounds may be lost because of irreversible adsorption on the column during the separation process. This phenomenon could be avoided by liquid–liquid extraction, which is often used to purify the crude extracts preliminarily in BGS. However, the low separation efficiency of traditional liquid–liquid extraction separation results in extracts with still very complex composition. Nevertheless, the liquid–liquid extraction method needs to be improved to obtain the target fraction.

High-speed counter-current chromatography (HSCCC), as a continuous liquid–liquid extraction method without any solid support, has been widely employed to prepare active compounds from crude extracts [10,11]. HSCCC can avoid the irreversible adsorption of samples on a solid phase. Compared with the traditional chromatography, HSCCC can improve the sample loading capacity and facilitate the large-scale preparation of active compounds. Accordingly, the impurities of the target fraction will be greatly reduced and the success rate of the separation of active compounds will be highly improved after separation by HSCCC.

Blueberries (*Vaccinium* spp.) are grown all over the world, especially in Northeast China. Blueberry cultivation is popular due to its attractive color, special fragrance, and high nutritional value [12]. Blueberry is rich in bioactive compounds, including anthocyanins, polyphenols, various antioxidant enzymes, and vitamins [13]. The bioactive compounds from blueberry have antioxidant, antitumor, and anti-inflammatory activities, as well as anti-aging effects [14]. Moreover, some bioactive compounds in blueberry could improve blood glucose and blood pressure levels [15].

α-Glucosidase (EC 3.2.1.20), as a hydrolytic enzyme, has the dual functions of hydrolysis and transglycosidation. Therefore, α-glucosidase is widely used in ethanol fermentation, oligosaccharide production, starch hydrolysis, food composition analysis, and metabolic mechanism research [16]. α-Glucosidase cleaves the 1,4-α glycosidic bonds of oligosaccharides/polysaccharides by adding water molecules. This catalytic reaction produces free α-D-glucose, which is absorbed by intestinal cells from the blood and results in postprandial hyperglycemia [17]. Controlling postprandial hyperglycemia is a critical method to control the incidence of type 2 diabetes (T2DM). α-Glucosidase has been used as a target enzyme to tackle the problems associated with T2DM. Some drugs treated T2DM by inhibiting the activity of α-glucosidase [18]. However, although these inhibitors are clinically effective, their use is limited due to adverse reactions. Hence, natural products are being tested as potential α-glucosidase inhibitors to avoid these problems. Currently, many studies have reported on the inhibitory effect of phenolic acids and flavonoids on α-glucosidase [19,20]. However, the components of blueberry with α-glucosidase inhibitory activity have not been elucidated yet. Few studies have reported on the interaction mechanism between blueberry components and α-glucosidase [17,21,22].

This study aimed to: (1) establish an efficient strategy for the selection of active components with α-glucosidase inhibitory activity based on counter-current fractionation and bioassay-guided separation; (2) investigate the interaction between the most active component and α-glucosidase in an aqueous solution by spectroscopic methods, including fluorescence and circular dichroism (CD) in combination with molecular docking techniques.

## 2. Materials and Methods

### 2.1. Material and Chemicals

Fresh Meden blueberries were collected from the Heilongjiang Sanmei Seedling Base (Shangzhi, Heilongjiang Province, China), and dried in a freezing-vacuum dryer (LGJ-10A, Shanghai Hefan Instrument Co., Ltd., Shanghai, China) at −18 °C until the pulp moisture content was less than 5%, and then the dehydrated pulp was milled into powdered particles using an electric mill (SG-1008, Changzhou Shuangge Drying Equipment Co., Ltd., Changzhou, Jiangsu Province, China). The powders were sieved using a 40-mesh screen and stored at 4 °C for future experiments.

α-Glucosidase (EC 3.2.1.20, Type I, 50 U/mg, 68.5 kDa) was produced from *Saccharomyces cerevisiae* and provided from Nanjing Sanshu Biotechnology Co., Ltd. (Nanjing, Jiangsu Province, China). Acarbose, *p*-nitrophenol (*p*NP), and *p*-nitrophenyl-α-D-glucopyranoside (*p*NP-G) were offered from Hubei Weideli Chemical Technology Co., Ltd. (Wuhan, Hubei Province, China). Phosphate buffer solution (PBS, pH 6.8) was obtained from Beijing Shengdong Technology Co., Ltd. (Beijing, China). Ethyl acetate, ethanol, 1-butanol, and light petroleum were from Tianjin Tianxin Fine Chemical Factory (Tianjin, China) and were analytical grade. Methyl tert butyl ether (MTBE), acetonitrile (ACN), and trifluoroacetic acid (TFA) were obtained from Jinan Guruite Chemical Co., Ltd. (Jinan, Shangdong Province, China).

### 2.2. Preparation of Sample

The experiment of ultrasonic assisted extraction was carried out with ultrasound equipment (YM-T2000CT, Shanghai Yuming Instrument Co., Ltd., Shanghai, China) with a maximum power of 1000 W and a bath volume of 50 L. The lyophilized blueberry powders (1000 g) were mixed with 60% ethanol (*v/v*) at a solid-to-liquid ratio of 1:3 g/mL. The ultrasound power (300 W), extraction time (20 min), and extraction temperature (45 °C) were set by the ultrasound equipment control panel. Each experiment was repeated three times. The extracts were combined and centrifuged (4000× *g* for 20 min) using a high-speed freezing centrifuge (3-18KS, Beijing Bomaixing Instrument Co., Ltd., Beijing, China). The filtrate was concentrated by using a vacuum rotary evaporator (DZFY-2L, Shanghai Xingke Instrument Co., Ltd., Shanghai, China) at 40 °C and freeze-dried via the freezing-vacuum dryer for 24 h to obtain the crude sample (46.38 g). The crude sample was dissolved in water and extracted three times successively with 500 mL light petroleum, ethyl acetate, and 1-butanol. After concentration and vacuum freeze-drying, 0.84 g of light petroleum extract (Sample 1), 3.11 g of ethyl acetate extract (Sample 2), 7.26 g of 1-butanol extract (Sample 3) and 21.73 g of water-soluble extract (Sample 4) were obtained.

### 2.3. HSCCC Fractionation Procedure

HSCCC separation was carried out on a model TBE-300V HSCCC system (Shanghai Tongtian Biotechnology Co., Ltd., Shanghai, China). 1-Butanol-MTBE-ACN-water-TFA (2:2:1:5:0.01, *v*/*v*/*v*/*v*/*v*) was used as the solvent of HSCCC [23]. The lower phase and the upper phase were used as the mobile phase and the stationary phase, respectively. The upper phase was initially filled the multilayer column entirely. The flow rate of the mobile phase and the rotating speed of the apparatus were 2.0 mL/min and 900 rpm, respectively. The system in the column reached the hydrodynamic equilibrium when the mobile phase flowed out from the tail outlet, and then the sample was injected through the injection valve. The UV-6100 detector (Shanghai Meipinda Instrument Co., Ltd., Shanghai, China) at 254 nm was used to continuously monitor the column effluent. Fractions were collected into test tubes and then evaporated by using a vacuum rotary evaporator at 40 °C. The stationary phase retention rate (*S_r_*) was calculated by the Equation (1):

(1) $$S_{r}=\frac{V_{s}}{V_{c}}\times 100\%$$

where vs. and *V_c_* are the stationary phase volume and the total column volume, respectively.

### 2.4. Sephadex LH-20 Column Chromatography

Sephadex LH-20 gel was employed to further isolate and purify the fraction with high α-glucosidase inhibitory activity obtained by HSCCC [24]. Sephadex LH-20 gel (20 g) was sufficiently saturated with 200 mL of distilled water, transferred to an eluting column (30 cm × 2.5 cm), and eluted with 45% acetone for twice the volume of the column. The fraction was filtered with a 0.22 μm microporous membrane, and 2 mL fraction was collected as a sample. After the fraction completely penetrated into the gel bed, the chromatographic column was eluted with 50% HCl-methanol solution (0.1% (*v*/*v*)). The eluent was collected, concentrated, and freeze-dried to obtain a high-purity fraction with high α-glucosidase inhibitory activity.

### 2.5. HPLC Analysis and Identification of Isolated Fraction

Sample 4, counter-current fractions and an isolated target compound with high inhibition α-glucosidase activity, was analyzed by using HPLC. In total, 5% (*v*/*v*) formic acid and 1% (*v/v*) formic acid acetonitrile were used as the mobile phases A and B, respectively. The gradient elution was as follows: 5%–20% B from 0 to 10 min, 20%–25% B from 10 to 15 min, 25%–30% B from 15 to 25 min, 30%–33% B from 25 to 30 min and 33%–5% B from 30 to 45 min. The flow rate of mobile phase and the injection volume of sample were set at 0.8 mL/min and 20 µL, respectively. In addition, the wavelength and column temperature were 520 nm and 25 °C, respectively. The purity of component (*w_i_*) could be calculated by using the equation 2 [25].

(2) $$w_{i}(\%)=\frac{f_{i}A_{i}}{\sum f_{i}A_{i}}$$

where *w_i_* and *A_i_* are purity and peak area of component *i*, respectively. *f_i_* is the correction factor.

Structure identification of the isolated compound with high α-glucosidase inhibitory activity was performed by its ultraviolet spectral, Fourier-transformed infrared (FTIR) spectroscopy, high-performance liquid chromatography-electrospray ionization-tandem mass spectrometry (HPLC-ESI-MS/MS), ^1^H-NMR, and ^13^C-NMR. The specific operation steps of the above methods are shown in the Supplementary Materials.

### 2.6. α-Glucosidase Inhibition Assay

The inhibitory effect of the crude sample, Samples 1–4, counter-current fractions, and isolated target compound on α-glucosidase was evaluated in this study. The inhibition rate of α-glucosidase was determined based on a previously published method [26]. α-Glucosidase can hydrolyze *p*NP-G to *p*NP, which has the maximum absorption at 405 nm. α-Glucosidase and *p*NP-G (1 μM) were prepared with 0.1 M PBS (pH 6.8). α-Glucosidase (0.2 μM, 1 mL) was separately mixed with 1 mL of the crude sample, Samples 1–4, counter-current fractions (10, 20, 40, 80, and 160 μg/mL), and isolated target compound solution (10, 20, 40, 80, and 160 μM). The mixtures were shaken in a test tube and incubated in a 37 °C water bath for 10 min. The control was prepared by using 1 mL of PBS (0.1 M, pH 6.8) to replace the sample. Then, 1 mL of *p*NP-G solution was added in each test tube. The mixture was incubated in a 37 °C water bath for 20 min. Finally, 1 mL of anhydrous methanol was added rapidly to the mixture to stop the reaction. The α-glucosidase activity of each sample was measured by determining the absorbance at 405 nm. In the blank tube, 1 mL sample was mixed with 1 mL enzyme and 1 mL buffer solution. Finally, 1 mL of anhydrous methanol was added rapidly to inactivate the enzyme. Half maximal inhibitory concentration (IC_50_) was used to compare the inhibitory effect of the samples. Inhibition rate was calculated according to the equation 3.

(3) $$\mathrm{Inhibition}\;\mathrm{rate}\;(\%)=\frac{A_{\mathrm{control}}-\left(A_{\mathrm{sample}}-A_{\mathrm{blank}}\right)}{A_{\mathrm{control}}}\times 100$$

### 2.7. Inhibitory Kinetics

The inhibitory kinetics of α-glucosidase by cyanidin-3-glucoside (C3G) was measured by the Lineweaver–Burk equation. The *p*NP-G in the concentration range of 0.2–1.0 mmol/L was used as substrate for α-glucosidase. The C3G concentrations (0, 40, 160 μM) and α-glucosidase (0.2 μM) were fixed. The reaction rate was measured under the above experimental conditions. The Lineweaver–Burk double reciprocal curve was obtained by plotting the reciprocal of *p*NP-G concentration (1/(*S*)) with the enzyme reaction rate (1/*V*) to determine the inhibition type of α-glucosidase.

### 2.8. Fluorescence Spectroscopy

Fluorescence measurements were preformed via a DSF-150WT fluorescence spectrometer (Hitachi, Japan). The final concentration of α-glucosidase was prepared to be 20 μM in all solutions, while the different C3G final concentrations in the solutions were 0, 10, 20, 40, 80, 160 μM, respectively. α-Glucosidase and C3G were mixed well. The excitation wavelength and excitation slit were 295 nm and 5 nm, respectively. The fluorescence spectra of each mixture was obtained in the range of 312–500 nm under three temperatures (298, 308, and 318 K).

### 2.9. Surface Hydrophobicity Measurements

The surface hydrophobicity was determined by a DSF-150WT fluorescence spectrometer with 1-anilinonaphthalene-8-sulfonic acid (ANS) as the hydrophobicity fluorescence probe. The excitation slit, emission slit, and excitation wavelength were 5.0, 2.5, and 390 nm, respectively [27]. Small aliquots (20 μL) of C3G solution (1.5 mM) were sequentially titrated into a protein-ANS solution (3.0 mL 2.0 μM α-glucosidase with 16 μL 8 mM ANS). The maximum added volume of C3G solution was 100 μL, which was negligible for slight dilution of the protein during titration. The surface hydrophobicity of the α-glucosidase-C3G mixed system was showed as the relative ANS-fluorescence intensity.

### 2.10. Circular Dichroism

The secondary structure change of α-glucosidase in the presence of C3G was determined by CD spectrum. Samples of α-glucosidase (20 μM) or α-glucosidase (20 μM) + C3G (50 μM) were used in this study. The spectra were recorded in the far UV range (200–250 nm) with a 1 mm quartz cell on a J-810 CD spectrophotometer (American Olis Company, Carlsbad, CA, USA). The setting conditions of CD spectra are as follows: The scanning rate of 60 nm/min, spectral resolution of 1 nm, response of 1 s, and band-width of 1 nm.

### 2.11. Molecular Docking

C3G was selected for molecular docking based on the above experimental analysis results. The molecular structure of C3G (CID: 132989527) was from the PubChem molecular library and employed for ligand docking. The crystal structure of α-glucosidase (PDB ID: 3A47) available in the RSCB Protein Data Bank database (https://www.rcsb.org/) was employed as a receptor in the docking procedure. AutoDck4.2 software was employed for docking simulation, and the possible conformations of ligand and macromolecule were calculated by using the Lamarck genetic algorithm. The optimal docking results were obtained and further analyzed based on the principle of minimum binding energy and the maximum number of junctions in the operation results.

## 3. Results and Discussion

### 3.1. Analysis of α-glucosidase inhibition results

**Figure 1:**
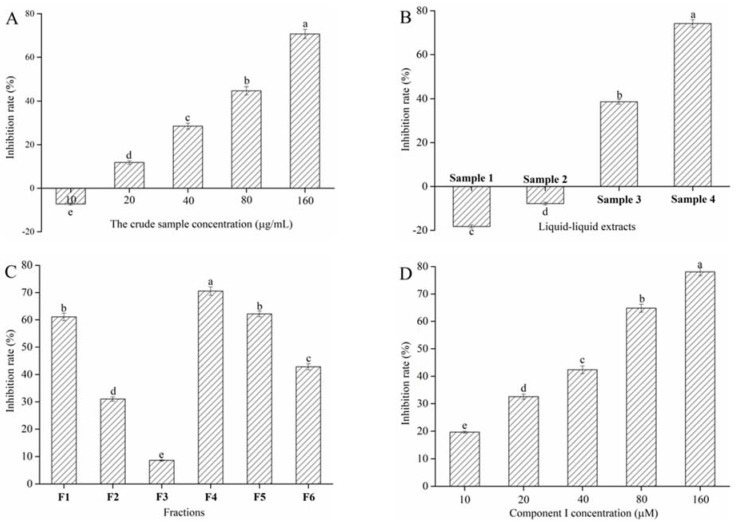
Effect of the crude sample (**A**), liquid–liquid extracts (**B**), counter-current fractions (**C**), and Cyanidin-3-glucoside (C3G) (**D**) from blueberry on α-glucosidase inhibition rate. Different lowercase letters indicate significant difference (*p* < 0.05).

Extraction and separation were performed under the guidance of α-glucosidase inhibitory activity assay to select for blueberry active compounds with α-glucosidase inhibitory activity. The crude sample could not inhibit α-glucosidase activity when its concentration was lower than 10 μg/mL (Figure 1A). However, the inhibitory rate obviously increased when the crude sample concentration increased from 20 to 160 μg/mL (*p* < 0.05) and reached inhibition equal to 70.69% ± 2.11% at 160 μg/mL. Results implied that the crude sample might contain active compounds with α-glucosidase inhibitory activity. Subsequently, the crude samples were extracted with light petroleum, ethyl acetate, and 1-butanol in turn. Figure 1B shows the inhibitory effect of different extracts on α-glucosidase activity at the concentration of 160 μg/mL. The results showed that Samples 1 and 2 could promote α-glucosidase activity. This phenomenon might be due to the fact that the active components of light petroleum and ethyl acetate extracts promoted more active sites of α-glucosidase to be exposed, which resulted in the increased activity of α-glucosidase. Nevertheless, Samples 3 and 4 could remarkably inhibit α-glucosidase activity, and the inhibitory effect of Sample 4 was significantly higher than that of Sample 3. Therefore, Sample 4 was selected for BGS.

### 3.2. HSCCC Fractions and Their Activity

**Figure 2:**
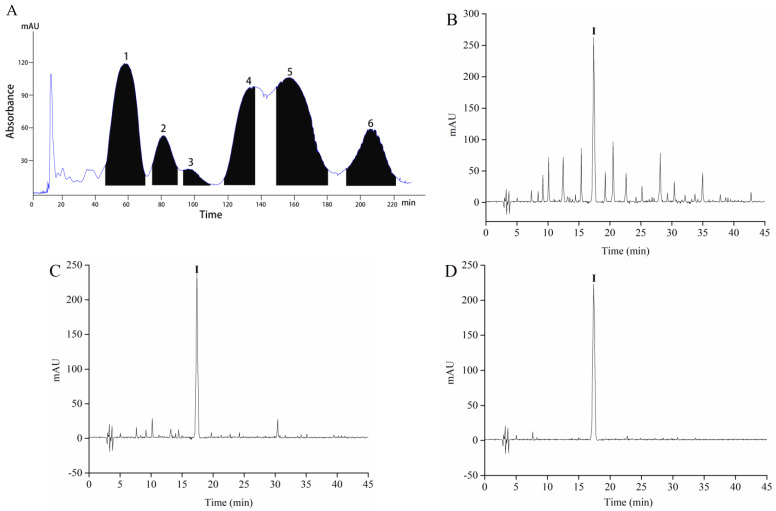
The High-speed counter-current chromatography (HSCCC) chromatogram of fractionation of Sample 4 (**A**), HPLC chromatograms of Sample 4 (**B**), F4 (**C**), and component I (**D**).

Sample 4 was separated by HSCCC to obtain different polar components. A two-phase solvent system that uses 1-butanol-MTBE-ACN-water-TFA was employed for HSCCC fractionation. HSCCC chromatogram of Sample 4 is shown in Figure 2A. According to the equation 1, the stationary phase retention rate was 70.06%. Six peak fractions (F1-6) were collected based on the chromatogram. The α-glucosidase inhibitory rate of F1-6 appears in Figure 1C. F4 shows a higher α-glucosidase inhibitory rate than the others when the peak fraction concentration was 160 μg/mL. Therefore, F4 was concentrated using a vacuum rotary evaporator at 40 °C, and a 28 mg sample was obtained from 1 g of Sample 4.

### 3.3. Isolation and Identification of Compound

**Figure 3:**
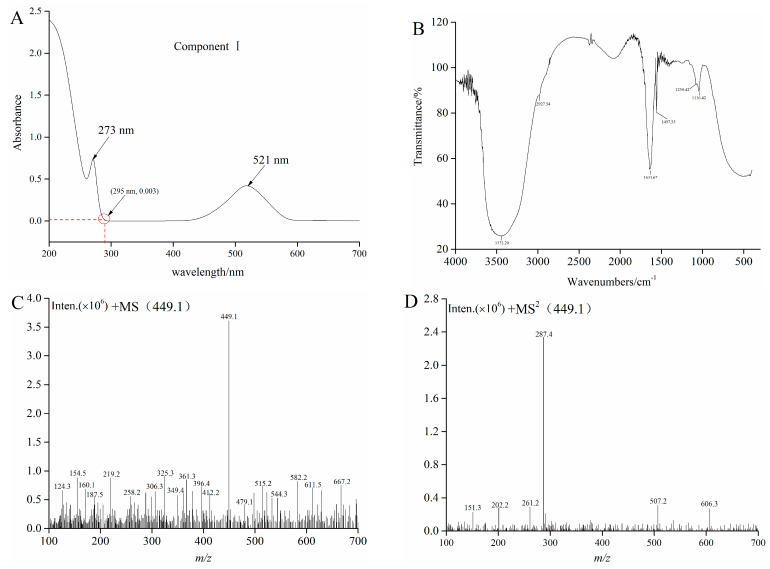
The UV (**A**), FTIR (**B**), MS (**C**), and MS^2^ (**D**) graphs of component I.

Sample 4 and F4 were initially analyzed by HPLC, and the chromatogram results are shown in Figure 2B,C. Sample 4 and F4 had many peaks, and results indicated that Sample 4 and F4 were the mixture. F4 contains one component with high content, which is recorded as component I. F4 was further purified by Sephadex LH-20 gel to obtain the high-purity component I. The purity of component I was 94.63% according to the equation 2. Then, ultraviolet spectral, FTIR spectroscopy, HPLC-ESI-MS/MS, and NMR were used to further analyze component I. Structure identification was performed by comparing the information of MS and NMR with the results described in the previous studies. The MS information and retention time of component I are shown in Table 1. The UV spectra, FTIR spectroscopy, and mass spectrogram of component I are displayed in Figure 3. ^1^H, ^13^C spectra, and NMR data of component I are shown in Figures S1 and S2, and Table S1.

As can be seen from Figure 3A, UV absorption peaks of component I appeared at about 280 nm and 520 nm. Results indicated that component I was an anthocyanins substance, and then the FTIR was employed to identify and confirm the functional groups [28]. The FTIR spectrum of component I in blueberry is shown in Figure 3B. The broad band at 3372 cm^−1^ might be due to the stretching vibration of O-H according to the obtained spectra, and the absorption band at 2927 cm^−1^ belonged to the asymmetric stretching of the C-H group. The band situated in the wave number 1631 cm^−1^ could be related to C=C scissoring vibration. In addition, infrared bands between 1497 cm^−1^ and 1116 cm cm^−1^ as “fingerprint” regions, such as those corresponding to the vibrations of C-O, C-C, and C-H, provided a considerable information about organic compounds, including alcohols, organic acids, and sugars [29]. Molaeafard et al. and Ma et al. used the FTIR analyses for identifying functional groups of anthocyanins in the extracts of sour cherry and black rice, respectively [30,31]. Subsequently, HPLC-ESI-MS/MS and NMR were used to further analyze component I. The MS and MS^2^ of component I (*t_R_* = 17.37) described *m/z* 449.1 and 287.4, respectively (Figure 3C,D). The fragment (M^+^-162) corresponds to glucose or galactose [24]. The MS^2^ fragment of the ion at m/z 287 is the characteristic ion of cyanidin. The results of the MS and NMR data of cyanide-3-glucoside in crude raspberry extract and *Liriope platyphylla* fruits by other authors were consistent with the results of this study [23,32]. Consequently, component I was cyanidin-3-glucoside (C3G).

α-Glucosidase inhibitory rate of a pure compound I (C3G) was assayed (Figure 1D). C3G could substantially inhibit α-glucosidase activity in a concentration-dependent manner (*p* < 0.05). The IC_50_ values of component I were 47.60 μM ± 0.92 μM. This phenomenon might be due to the interaction between the unsubstituted phenolic groups of C3G and α-glucosidase, which inhibited the α-glucosidase activity [33]. Hence, C3G was selected for subsequent experiments to confirm the reason for the above experimental results.

### 3.4. Inhibitory Kinetics of C3G on α-Glucosidase

**Figure 4:**
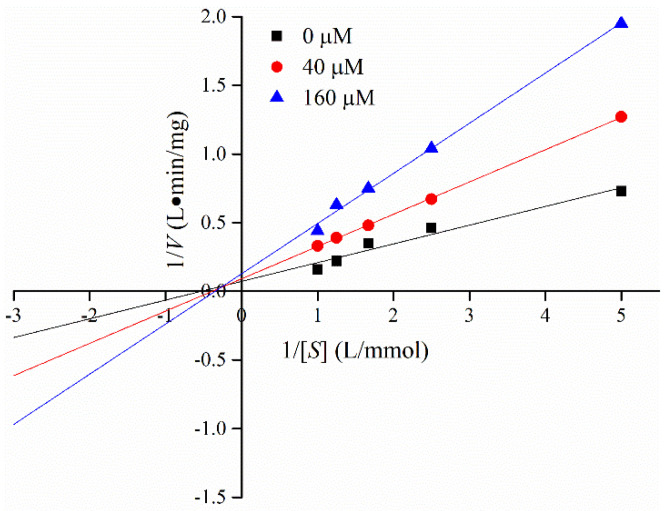
Lineweaver–Burk plot for determining the kinetic constants for α-glucosidase.

Lineweaver–Burk plots in the presence and absence of C3G were obtained at different substrate concentrations [*S*] to determine the interaction mechanism between C3G and α-glucosidase. Figure 4 shows the good linear relationship between 1/*S* and 1/*V* in the double reciprocal graph. Michaelis constant (*K_m_*) values increased, whereas the maximum reaction velocity (*V_m_*) decreased with the increase C3G concentration (Table 2). Results indicated that the inhibitory pattern of C3G on α-glucosidase was a combination of competitive and noncompetitive inhibition; that is, C3G could competitively interact with the binding site of the substrate and other active sites. The value of inhibition constant (*K_i_*) was calculated by the Dixon method. The *K_i_* value of α-glucosidase was 0.20 × 10^−3^ mmol/L. Different natural flavonoids have different inhibitory effects on α-glucosidase, and the inhibition modes of natural flavonoids on α-glucosidase were noncompetitive and anti-competitive [34].

### 3.5. Fluorescence Emission Spectroscopy

The interaction between α-glucosidase and C3G was studied by fluorescence emission spectroscopy. α-Glucosidase is composed of 589 amino acid residues and 20 tryptophan (Trp) residues based on the amino acid sequence of α-glucosidase from *Saccharomyces cerevisiae* with NCBI, which could provide intrinsic fluorescence. Consequently, the intrinsic fluorescence of α-glucosidase was measured at the excitation wavelength of 295 nm. The inner-filter effect is a practical challenge in fluorescence spectroscopy measurement because excitation/emission radiation is absorbed or scattered by substances in the surrounding solution [35]. Figure 3A displays that C3G showed very low absorption at the excitation wavelength (295 nm) and emission wavelength (339 nm). Therefore, the internal filter effect caused by C3G could also be ignored.

**Figure 5:**
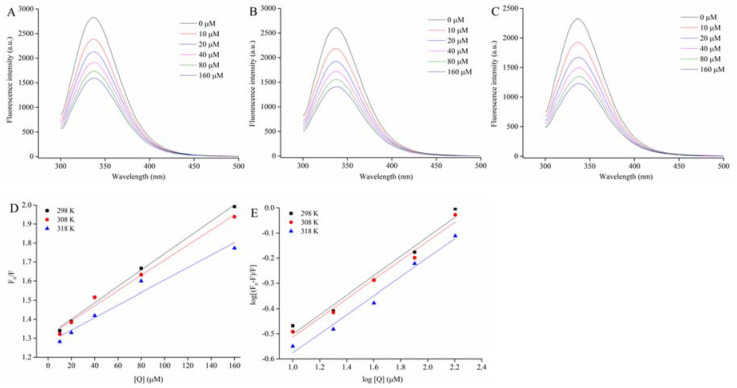
Effect of C3G on the intrinsic fluorescence spectrum of α-glucosidase at 298 (**A**), 308 (**B**), and 318 K (**C**); the Stern–Volmer plots for the quenching of α-glucosidase by C3G and the *K_q_* of α-glucosidase-C3G complex in 298, 308, and 318 K, respectively (**D**); the plots for the static quenching of α-glucosidase by C3G (**E**).

The fluorescence emission spectra of α-glucosidase in the presence of different C3G concentrations (0–160 μM) at three temperatures (298, 308, and 318 K) are shown in Figure 5A–C. The fluorescence intensity of α-glucosidase gradually quenched with the increase C3G concentration under three temperatures, without any distinct shift at the maximum absorption peak of α-glucosidase (about 338 nm). The intrinsic fluorescence quenching might be attributed to the interaction between C3G and α-glucosidase [36]. Previous researchers have confirmed that procyanidin dimer and kaempferol were able to quench the intrinsic fluorescence of α-glucosidase [37,38]. In addition, other studies have shown that C3G could quench the intrinsic fluorescence of some proteins, including hemoglobin, bovine serum albumin, myoglobin, and endothelial nitric oxidesynthase [39,40]. To sum up, these results support the conclusion that C3G interacts with α-glucosidase.

### 3.6. Fluorescence Quenching Mechanism and Binding Constant

The fluorescence data at three temperatures were analyzed by using the Stem–Volmer equation to determine the fluorescence quenching mechanism of α-glucosidase combined with C3G [41].

(4) $$F_{0}/F=1+K_{SV}\left[Q\right]=1+K_{q}\tau_{0}\left[Q\right]$$

where *F*_0_ and *F* are the fluorescence intensities without and with D3G, respectively. *K_SV_* is the Stem–Volmer quenching constant. *K_q_* and [*Q*] are the rate constant and C3G concentration of the bimolecular quenching process, respectively. *τ*_0_ is the average lifetime of fluorescence molecules without any quenching agent, 10^−8^ s. Figure 5D shows that the Stem–Volmer plots of α-glucosidase fluorescence quenched by the different C3G concentrations at three temperatures presented a good linear relationship (*R*^2^ > 0.95). Results demonstrated a single quenching mechanism (static or dynamic) [42]. The values of *K_SV_* and *K_q_* were calculated by using equation 4 and results are listed in Table 3. During the interaction of α-glucosidase and C3G, the values of *K_SV_* gradually decreased with the increased temperature. Results suggested that the quenching process was static quenching caused by the binding of C3G-α-glucosidase. Moreover, the values of *K_q_* were far greater than the maximum dynamic quenching constant values 2 × 10^10^ M^−1^∙s^−1^. Results further implied that C3G could effectively quench the intrinsic fluorescence of α-glucosidase by static quenching owing to the formation of α-glucosidase-C3G complexes.

For static quenching, the double logarithmic Stem–Volmer equation was determined by the binding constant (*K_a_*) and the number of binding sites (*n*) [43].

(5) $$\log\left[\left(F_{0}-F\right)/F\right]=\log K_{a}+n\log\left[Q\right]$$

The values of *K_a_* and *n* at each corresponding temperature are given in Table 3. The *K_a_* values were on the order of 10^5^ (Table 3). Results suggested that C3G had a strong binding affinity with α-glucosidase. The *K_a_* values of α-glucosidase-C3G decreased with the increase temperature, indicating that the complexes of α-glucosidase-C3G became unstable with the rising temperature. Moreover, the *n* values were approximately equal to 1 (Table 3), which illustrated the presence of one binding site in α-glucosidase for C3G during their interactions.

### 3.7. Thermodynamic Parameters

According to the fluorescence measurement results at 298, 308, and 318 K, the thermodynamic parameters, including Δ*H*, Δ*S*, and Δ*G*, were obtained to provide more information on the formation forces of C3G-α-glucosidase complexes. The main interactions between proteins and polyphenols were hydrophobic interactions, hydrogen bonding, van der Waals forces, and electrostatic interactions [44]. To investigate the interaction forces of C3G with α-glucosidase, the Van’t Hoff equation was employed to calculate the thermodynamic parameters [42].

(6) $$\ln K_{a}=-\frac{\Delta H}{RT}+\frac{\Delta S}{R}$$

(7) ΔG=ΔH−TΔS

where *K_a_* is the binding constant. The Δ*G* values of α-glucosidase-C3G at 298, 308, and 318 K were −3.44 ± 0.03, −2.46 ± 0.02, and −1.48 ± 0.03 kJ∙mol^−1^, respectively (Table 3). The negative Δ*G* values indicated that the binding reactions of α-glucosidase with C3G were spontaneous. In addition, the Δ*H* value of α-glucosidase-C3G was −32.64 ± 0.57 kJ∙mol^−1^. Results indicated that the binding reactions were exothermic. The evaluation principles of the interaction forces between protein and small molecule were obtained based on previous studies. The basic principles are as follows: (1) If Δ*H* > 0 and Δ*S* > 0, hydrophobic interaction is the main driving force. (2) If Δ*H* > 0 and Δ*S* < 0, hydrophobic and electrostatic interactions are the principal forces. (3) If Δ*H* < 0 and Δ*S* < 0, the hydrogen bonds between protein and ligand are the main forces. (4) If Δ*H* < 0 and Δ*S* > 0, electrostatic interactions are the fundamental forces. Δ*H* and Δ*S* values −32.64 kJ·mol^−1^ ± 0.57 kJ·mol^−1^ and −0.098 kJ·mol^−1^ ± 0.003 kJ·mol^−1^·K^−1^, respectively. Results indicated that C3G could interact with the aromatic amino acid residues (Trp) of α-glucosidase via the hydrogen bonds, which was consistent with the results where the main driving force of the interaction between procyanidin dimer and α-glucosidase was the hydrogen bonds [37]. The subsequent molecular docking simulation further supported the analysis results.

### 3.8. Protein Surface Hydrophobicity

**Figure 6:**
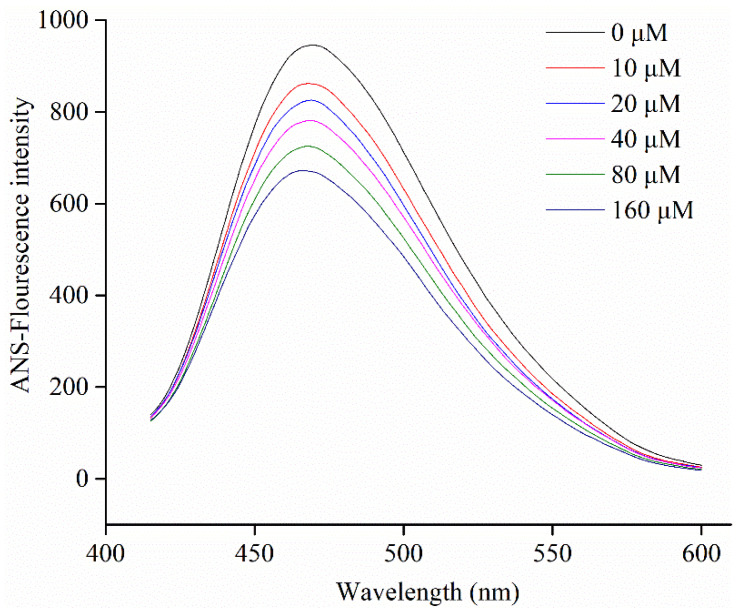
Changes in ANS-fluorescence intensity of α-glucosidase at different concentrations of C3G.

ANS, as a common hydrophobic probe, is employed to determine changes in the surface hydrophobicity of molecules [45]. The surface hydrophobicity of α-glucosidase in the presence of different levels of C3G was determined by a fluorescence spectrofluorimeter. Figure 6 shows that the surface hydrophobicity of α-glucosidase decreased when C3G concentration increased from 0 to 160 μM. Compared to the α-glucosidase alone, the surface hydrophobicity of α-glucosidase-C3G complex decreased, which could be explained by the binding of some C3G molecules to the hydrophobic groups on the surface of protein molecules [17].

### 3.9. Circular Dichroism Analysis

**Figure 7:**
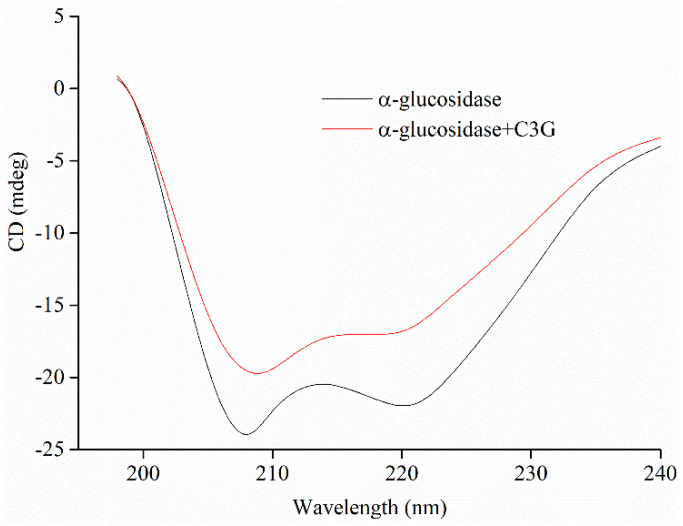
The profiles of α-glucosidase (20 μM) secondary structure treated by 50 μM C3G.

The conformational changes of the secondary structure of α-glucosidase in the absence and presence of C3G were quantitatively analyzed by CD. The CD spectra and the results of spectral analysis are shown in Figure 7 and Table 4, respectively. The CD spectra of α-glucosidase in the absence and presence of C3G showed two negative bands at about 209 and 221 nm, which are the typical features of α-helix structure. Figure 7 shows that the addition of C3G increased the values of the negative ellipticity, which indicated that the secondary structure of α-glucosidase partially changed [46,47]. The structural contents of α-glucosidase were calculated using the CD Pro software. In the presence of C3G, the α-helical and β-turn contents of α-glucosidase markedly decreased to 19.3% ± 0.5% and 20.8% ± 0.4%, respectively. Nevertheless, the β-sheet content of α-glucosidase and the irregular coil structures increased to 26.3% ± 0.4% and 35.0% ± 0.4%, respectively. The change in the secondary structure of α-glucosidase might be due to the promotion of the unfolding of the protein polypeptide chain and the destruction of the hydrogen bond network structure of α-glucosidase owing to the binding between C3G and α-glucosidase [34]. This unfolding resulted in the less compact structure of α-glucosidase.

### 3.10. Molecular Docking Analysis

**Figure 8:**
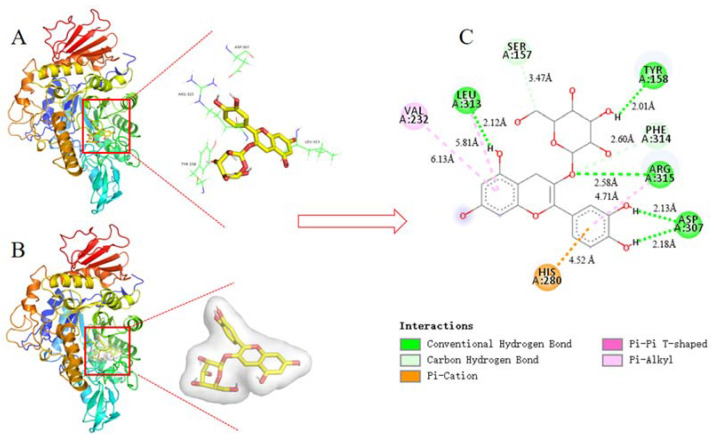
The 3D structure of α-glucosidase including active amino acid and docking active site (**A**); 3D docking mode between C3G and α-glucosidase simulated by Discovery Studio and the amino acid of active site (**B**); 2D schematic interaction diagram between C3G and α-glucosidase, the color of amino acid residue is drawn by interaction (**C**). The hydrogen bonds interaction between C3G and the amino acid residue of α-glucosidase is shown in green, and the hydrophobic interactions between C3G and the amino acid residue of α-glucosidase are described in purple, light purple, and orange.

C3G is an important anthocyanin component with the highest α-glucosidase inhibitory activity in blueberry extract based on the above research and analysis. Thus, C3G was selected for docking with α-glucosidase to define the best binding sites and confirm the experimental results. After 100 docking operations, the one with the lowest energy and the most number of combinations from the docking results in AutoDock was selected as the final analysis results. The binding energy of the complex (C3G-α-glucosidase) was -11.71 kcal·mol^−1^. Results were consistent with the estimated binding energy, indicating that the result of the molecular docking analysis was accurate and feasible. The combined 3D docking mode (Figure 8A,B) and 2D schematic diagram (Figure 8C) clearly showed that C3G was inserted into the active pocket of α-glucosidase. Figure 8C displays that C3G interacted with amino acid residues, including Ser 157, Tyr 158, Phe 314, Arg 315, Asp 307, His 280, Val 232, and Leu 313, which were the possible interaction sites between C3G and α-glucosidase. Seven hydrogen bonds were found between C3G and the amino acid residues (Leu 313, Ser 157, Tyr 158, Phe 314, Arg 315, and two Asp 307) because C3G has many hydroxyl groups. Four hydrophobic interactions (one Pi-Pi T-Shaped, one Pi-Cation, and two Pi-Alkyl) were found between C3G and the amino acid residues (Val 232, Leu 313, Arg 315, and His 280). These results indicated that α-glucosidase had different C3G binding sites, which explained the differences in the secondary structure between α-glucosidase and C3G. Tyr and Phe have intrinsic fluorescence absorption properties. C3G and amino acid residues of α-glucosidase (Tyr and Phe) formed complexes by hydrogen bond force (Figure 8C). This result better explained the reason why the interaction between C3G and α-glucosidase reduced the fluorescence intensity of α-glucosidase. The distances of seven hydrogen bonds (2.12 Å, 3.47 Å, 2.01 Å, 2.60 Å, 2.58 Å, 2.13 Å, and 2.18 Å) and the distances of four hydrophobic forces (6.18 Å, 5.81 Å, 4.71 Å, and 4.52 Å) are shown in Figure 8C. Results of the comprehensive comparison of the interactions of C3G and α-glucosidase showed that the main driving force of the interaction between C3G and α-glucosidase was hydrogen bonds. This finding further proved the results of fluorescence spectroscopy (thermodynamic parameter). Figure 8A shows that C3G bound to the active sites of α-glucosidase, including Leu 313, Ser 157, Tyr 158, Phe 314, Arg 315, and two Asp 307. Tyr 158 interacted with C3G via hydrogen bonds and thus inhibited the activity of α-glucosidase.

## 4. Conclusions

Cyanidin-3-glucoside (C3G) with a purity of 94.16% was successfully obtained from blueberry based on counter-current fractionation and bioassay-guided separation. In addition, the obtained findings explicitly characterized and described the interaction mechanism between α-glucosidase and C3G using spectroscopic analyses and molecular docking. C3G could spontaneously bind with α-glucosidase to form complexes by hydrogen bonds. The secondary structure of glucosidase changed in varying degrees after complexation with C3G. The α-helical and β-turn contents of α-glucosidase decreased, whereas the β-sheet content of α-glucosidase and the irregular coil structures increased. These findings provide a new insight into the inhibitory mechanism of α-glucosidase on C3G.

## Figures and Tables

**Table 1:** Identify of the component I using HPLC-ESI-MS/MS.

Peaks	Retention Time (min)	Molecular Ion (*m/z*)	Molecular Fragments (*m/z*)	Lost Fragments (*m/z*)	Compound
Component I	17.37	449.1	287.4	162	Cyanidin-3-glucoside

**Table 2:** *V_max_* and *K_m_* values of the interaction between C3G and α-glucosidase.

Enzyme	Inhibitor	*V_max_*	*K_m_*	*K_i_*
		(mg∙L^−1^∙min^−1^)	(mmol∙L^−1^)	(mmol∙L^−1^)
α-Glucosidase	Non	13.78	1.88	0.20 × 10^−3^
	C3G (40 μM)	10.97	2.57	
	C3G (160 μM)	7.86	2.87	

**Table 3:** The values of quenching and binding parameters for C3G binding with α-glucosidase at different temperatures (298, 308, and 318 K).

Sample	T (K)	*K_sv_* (10^3^ M^−1^)	*K_q_* (10^11^ M^−1^∙S^−1^)	*K_a_* (10^5^ M^−1^)	n	*R* ^2^	Δ*H* (kJ∙mol^−1^)	Δ*S* (kJ∙mol^−1^∙K^−1^)	Δ*G* (kJ∙mol^−1^)
α-Glucosidase	298	4.29 ± 0.02	4.29 ± 0.02	1.30 ± 0.01	0.88	0.9930	−32.64 ± 0.57	−0.098 ± 0.003	−3.44 ± 0.03
	308	3.97 ± 0.03	3.97 ± 0.03	1.27 ± 0.03	0.89	0.9833			−2.46 ± 0.02
	318	3.28 ± 0.01	3.28 ± 0.01	1.11 ± 0.02	0.95	0.9552			−1.48 ± 0.03

**Table 4:** Secondary structure analysis of α-glucosidase and its complex with C3G.

Sample	α-Helix (%)	β-Sheet (%)	β-Turn (%)	Irregular Coil (%)
α-Glucosidase	22.7 ± 0.4 ^a^	23.5 ± 0.2 ^a^	24.1 ± 0.3 ^a^	32.4 ± 0.2 ^b^
α-Glucosidase-C3G	19.3 ± 0.5 ^b^	26.3 ± 0.4 ^b^	20.8 ± 0.4 ^b^	35.0 ± 0.4 ^a^

Different the lower case letters indicate significant difference (*p* < 0.05).

## Supplementary Materials

The following are available online at https://www.mdpi.com/2304-8158/10/3/509/s1, the detailed processes of UV spectra, Fourier-transformed infrared (FTIR) spectroscopy, HPLC-ESI-MS/MS, ^1^H-NMR, and ^13^C-NMR, Figure S1 ^1^H NMR (400 MHz, CD3OD) spectrum of compound I, Figure S2 ^13^C-NMR (100 MHz, CD3OD) spectrum of component I, Table S1: ^1^H, ^13^C-NMR data for component I in CD3OD.
