# Supplementary material for: Counter-Current Fractionation-Assisted Bioassay-Guided Separation of Active Compound from Blueberry and the Interaction between the Active Compound and α-Glucosidase
Source: Foods. 2021 Mar 1;10(3):509. doi: 10.3390/foods10030509 (PMC7998573; doi:10.3390/foods10030509)
Supplement: Supplementary file 1 [file foods-10-00509-s001.pdf]

Table of Contents

CONTENTS	page
The detailed processes of UV spectra, Fourier-transformed infrared (FTIR) spectroscopy, HPLC-ESI-MS/MS, ^{1}H-NMR, and ^{13}C-NMR	2-3
**Table S1** ^{1}H, ^{13}C-NMR data for component I in CD_3OD	4
**Figure S1** ^{1}H NMR (400 MHz, CD_3OD) spectrum of compound I	5
**Figure S2** ^{13}C-NMR (100 MHz, CD_3OD) spectrum of component I	6

The detailed processes of UV spectra, Fourier-transformed infrared (FTIR) spectroscopy, HPLC-ESI-MS/MS, ^{1}H-NMR, and ^{13}C-NMR are as follows:

Ultraviolet spectral scanning of C3G

The UV spectrum of C3G was obtained using a UV-visible spectrophotometer (UV1800, Qingdao Power Weiye environmental protection equipment Co., Ltd, Qingdao, Shandong Province, China). In brief ,100 μL of C3G solution (3.547 mM) was added into 3 mL of PBS. The absorption spectrum of C3G was recorded from 200 to 700 nm.The absorption spectrum for a PBS blank was subtracted from that of the sample. The UV measurements were used to analyze the inner filter effects of the C3G.

Fourier-transformed infrared (FTIR) spectroscopy

FTIR was performed using a FTIR spectrophotometer. Component I (5 mg) was mixed completely with KBr (~1g) and pellets were formed. The sample pellets were then analysed using an FTIR spectrophotometer and spectra were recorded in the 4000−500 cm^{-1} region. FTIR analyses were carried and the graph was plotted with the help of Origin version 9.0 software.

HPLC-ESI-MS/MS

Component I was characterised by HPLC-ESI-MS/MS. A sample (5 μL) of component I was injected into an analytical reverse phase C-18 column (4.6 mm × 150 mm, 5 μm, Agilent). 5% (*v*/*v*) formic acid and 1% (*v*/*v*) formic acid acetonitrile were used as the mobile phases A and B, respectively. The gradient elution was as follows: 5%-20% B from 0 to 10 min, 20%-25% B from 10 to 15 min, 25%-30% B from 15 to 25 min, 30%-33% B from 25 to 30 min and 33%-5% B from 30 to 45 min. The mass spectra (MS) were obtained at a range of 100-1000 m/z in the positive mode. The voltage of capillary was set 2.0 kV. The voltages of sampling cone and extraction cone were 40

V and 2.0 V, respectively. The collision energy was 20.0-45.0 eV. The time of scan and interscan were 13 min and 0.28 s, respectively. Mass-Lynx TM V 4.1 software was employed to analyse experimental results.

NMR

^{1}H and ^{13}C-NMR spectra were recorded on a Bruker Advance III 400 spectrometer (Bruker, Switzerland). Component I was dissolved in CD_3OD. The structure of component I was identified by comparing the experimental results with those reported in the literature.

Table 1. ^{1}H-NMR (400 MHz), ^{13}C-NMR(100 MHz) data for component Ⅰ in CD_3OD.

Position	Component I	
	^{1}H-NMR	^{13}C-NMR
2		162.8
3		144.2
4	9.03 (1H, s)	134.6
5		157.8
6	6.67 (1H, d, *J* =1.8 Hz)	102.2
7		168.9
8	6.91 (1H, d, *J* =1.8 Hz)	93.6
9		156.2
10		115.9
1′		119.8
2′	8.04 (1H, d, *J* =1.9 Hz)	116.9
3′		146.0
4′		153.4
5′	7.02 (1H, dd, *J* =8.8, 1.9 Hz)	111.9
6′	8.28 (1H, d, *J* = 8.8, 1.9 Hz)	126.9
1′′	5.33 (1H, d, *J* =7.7 Hz)	101.8
2′′		73.3
3′′	3.94~3.47 (6H, m)	76.7
4′′		69.6
5′′		77.4
6′′		60.9
1′′′		
2′′′		
3′′′		
4′′′		
5′′′		
6′′′		

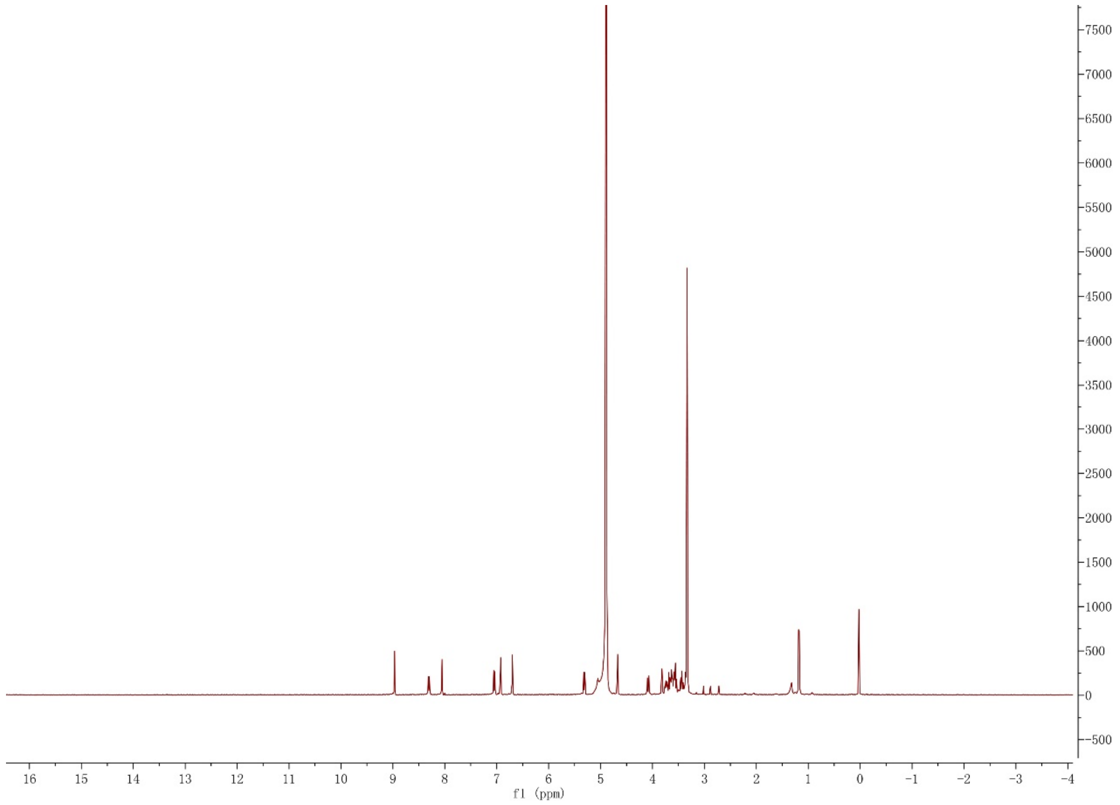

Figure S1

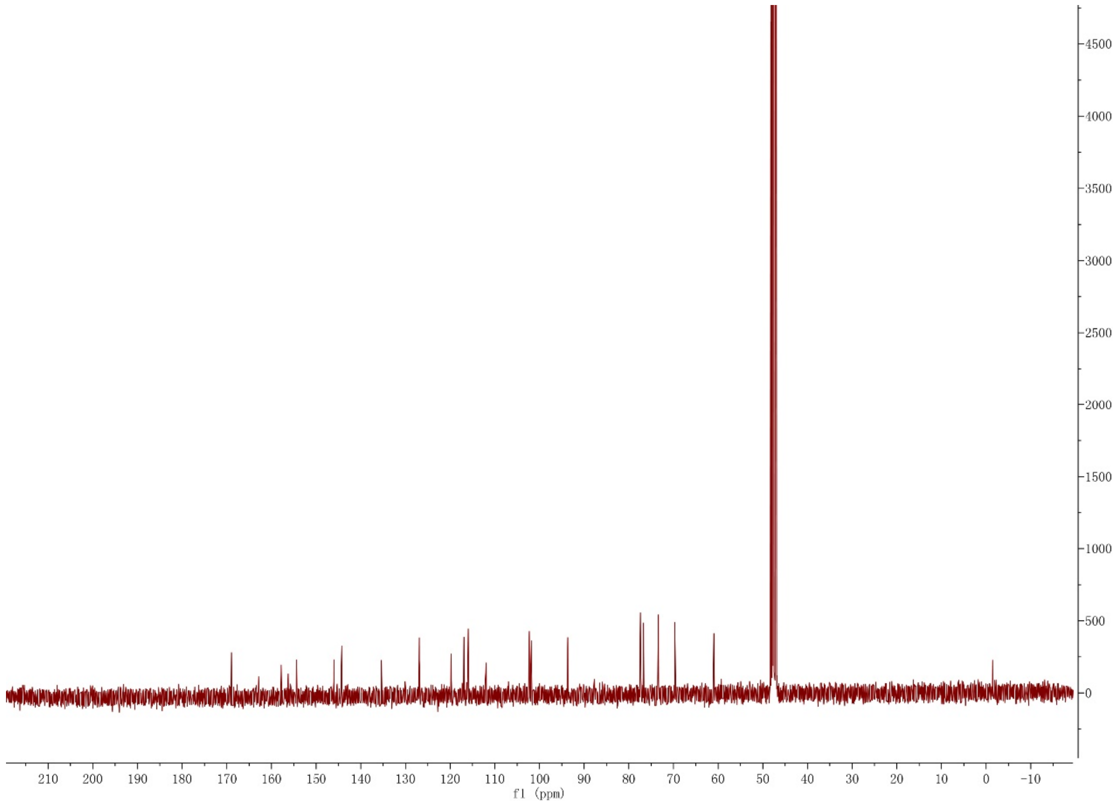

Figure S2
